# Structure of the tsunamigenic plate boundary and low-frequency earthquakes in the southern Ryukyu Trench

**DOI:** 10.1038/ncomms12255

**Published:** 2016-07-22

**Authors:** Ryuta Arai, Tsutomu Takahashi, Shuichi Kodaira, Yuka Kaiho, Ayako Nakanishi, Gou Fujie, Yasuyuki Nakamura, Yojiro Yamamoto, Yasushi Ishihara, Seiichi Miura, Yoshiyuki Kaneda

**Affiliations:** 1 Research and Development Center for Earthquake and Tsunami, Japan Agency for Marine-Earth Science and Technology, 3173-25 Showa-machi Kanazawa-ku, Yokohama, Kanagawa 236-0001, Japan; 2 Institute of Education, Research and Regional Cooperation for Crisis Management Shikoku, Kagawa University, 1-1 Saiwai-cho, Takamatsu, Kagawa 760-8521, Japan

## Abstract

It has been recognized that even weakly coupled subduction zones may cause large interplate earthquakes leading to destructive tsunamis. The Ryukyu Trench is one of the best fields to study this phenomenon, since various slow earthquakes and tsunamis have occurred; yet the fault structure and seismic activity there are poorly constrained. Here we present seismological evidence from marine observation for megathrust faults and low-frequency earthquakes (LFEs). On the basis of passive observation we find LFEs occur at 15–18 km depths along the plate interface and their distribution seems to bridge the gap between the shallow tsunamigenic zone and the deep slow slip region. This suggests that the southern Ryukyu Trench is dominated by slow earthquakes at any depths and lacks a typical locked zone. The plate interface is overlaid by a low-velocity wedge and is accompanied by polarity reversals of seismic reflections, indicating fluids exist at various depths along the plate interface.

## Abstract (web summary)

Weakly coupled subduction zones may generate earthquakes that lead to tsunamis, but their structure and seismicity are poorly constrained with the Ryukyu subduction zone as one such example. Here, Arai *et al*. present seismological evidence from Ryukyu showing megathrust faults and low frequency earthquakes.

## Graphical abstract

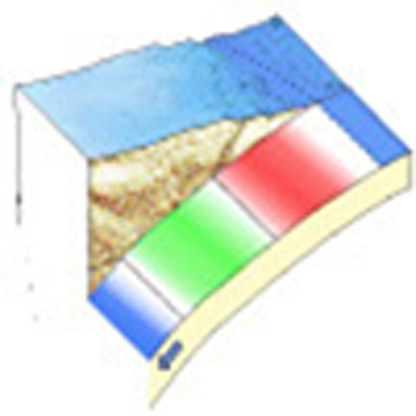

The Ryukyu subduction zone, extending 1,200 km from Kyushu, SW Japan, to Taiwan has been intensively examined in terms of its seismic and tsunami potentials^1^,^2^,^3^. The Ryukyu subduction zone has lacked clear evidence for great interplate earthquakes (M>8) in the last few hundred years and thus the overall plate coupling is thought to be weak^4^. The weak coupling is also supported by recent geophysical/geological studies: First, seismic studies reveal that slow slip events (SSEs), very-low-frequency earthquakes (VLFEs) and small repeating earthquakes are ubiquitously distributed, suggesting that the plate boundary is enriched in fluids and mostly creeping^5^,^6^,^7^,^8^,^9^. Second, geodetic observations indicate that at the central Ryukyu Trench a possible coupled zone is very narrow and that, if any, it is limited to the shallowest part of the plate boundary^10^. Third, the Yaeyama earthquake in 1771, which is thought to have ruptured a shallow portion of the plate interface^11^ and generated a huge tsunami with a maximum run-up height of ∼30 m (ref. 12), may have been a tsunami earthquake since it has a large discrepancy of its earthquake magnitude and tsunami magnitude^13^,^14^. Recent studies of tsunami deposits indicate that the Ryukyu Islands have been periodically hit by large tsunamis with an interval of several hundred years^15^. This may indicate that the Ryukyu Trench has repeatedly generated such tsunami earthquakes, which usually occur along a stably sliding zone with weak coupling^16^,^17^,^18^. Therefore, the Ryukyu subduction zone is one of the best fields to study how subduction zones with weak coupling works. However, all of the previous evidences mentioned above have been obtained by extremely sparse land observation network and have insufficient resolution to discuss the spatial variation and segmentation in frictional property at the plate boundary. Especially in the southern part of the Ryukyu Trench, there is a gap between the shallow tsunamigenic zone (source region of Yaeyama earthquake tsunami in 1771 (ref. 11)) and the deep source region of repeating SSEs^5^. Generally, this kind of gap corresponds to a seismogenic zone in other areas^19^, but the coupling condition there in the Ryukyu subduction zone remains enigmatic.

It has been recognized that slow earthquakes including LFE, VLFEs and SSEs are a key to understand the frictional properties along the plate interface^19^. Generally, these anomalous events occur in the downdip limit of seismogenic zones or along the shallow plate boundaries or branching faults within the frontal accretionary wedge^20^,^21^. Seismic signals from these events have characteristic features of a longer duration and are enriched in low-frequency energy (for example, lower than 10 Hz) and depleted in higher frequencies compared with regular earthquakes. It is also known that LFEs and VLFEs are often coincident with episodic tremors and SSEs both in space and time^22^,^23^. Although their locations are an important information, slow earthquakes are difficult to detect and locate due to their small amplitude and the lack of clear onsets of the seismic waves. Recent seismic studies show that the source regions of slow earthquakes are closely correlated with the surrounding structure: They typically occur in the regions with low seismic velocities where high pore fluid pressure is expected^24^. In the Ryukyu subduction zone, however, such structural characteristics have not been examined in detail. In addition, megathrust fault system and its relation to the distribution of slow earthquakes are unknown due to the lack of close-in observations.

To reveal fine-scale structure of subduction faults and their frictional property in the southernmost part of the Ryukyu subduction zone, we carried out integrated seismic experiments in 2013. In this region, the Philippine Sea plate with ages from 49 Ma to 33 Ma is subducting beneath the Eurasian plate at a rate of over 10 cm per year^25^,^26^. To record seismic signals, we deployed 30 ocean bottom seismographs (OBSs) on the seafloor at 20–30 km interval for 3 months. We also used broadband and short-period seismometers at six on-land temporary stations. For refraction studies, we laid out 60 OBSs at 6 km interval on a ∼390-km-long line perpendicular to the trench axis and obtained a two-dimensional (2D) P-wave velocity image with seismic reflectors. On the same line, we conducted a 2D multichannel seismic (MCS) reflection survey.

Using this data set, we demonstrate that the plate interface in the southern Ryukyu trench is dominated by slow earthquakes at any depths and lacks a typical locked zone. Moreover, we find megathrust fault system including a branching fault coincide with a potential source region of tsunami earthquakes.

## Results

### Detection of LFEs and their relation to fault structure

**Figure 1:**
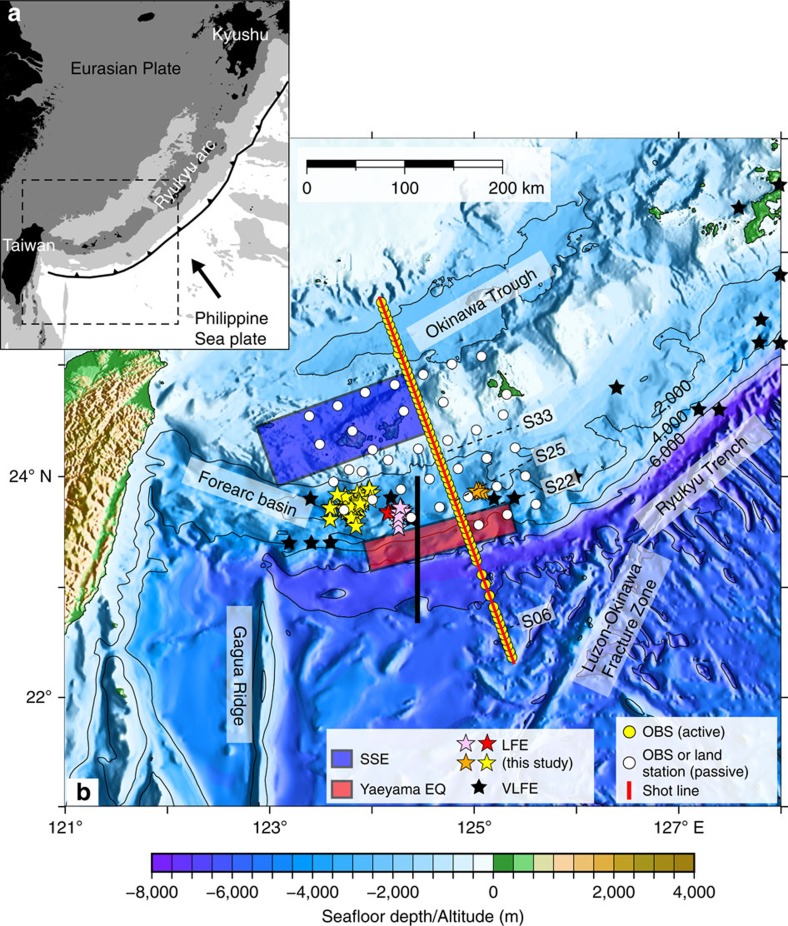
Layout of the seismic experiment. (**a**) Regional tectonic map of the Ryukyu subduction zone. Areas above sea level is shaded black, and seafloors shallower than 1,000 and 4,000 m below sea level are shaded in dark and light grey, respectively. Black arrow indicates the convergence direction of the Philippine Sea plate with respect to the Eurasian plate^26^. (**b**) Bathymetric map of the southern Ryukyu trench. Background colours represent seafloor depth in metre, with contours every 2,000 m. Yellow and white circles indicate the positions of ocean bottom seismographs (OBSs) for active and passive sources, respectively. Airgun shots occurred on the red line. Low-frequency earthquakes (LFEs) detected by this study (coloured stars) consist of four sequences and their colours correspond to different sequences of LFEs (sequence A–D). Black stars indicate the epicentres of very-low-frequency earthquakes (VLFEs) from ref. 6. The source regions of the Yaeyama earthquake in 1771 (ref. 11) and repeating slow slip events (SSEs)^5^ are shown by red and blue square, respectively. Comparison of our reflection data with the TAIGER project^28^ (thick black line) are presented in Supplementary Fig. 3. Seismic records of numbered OBSs for refraction studies (S06, S22, S25 and S33) are presented in Supplementary Fig. 4.

**Figure 2:**
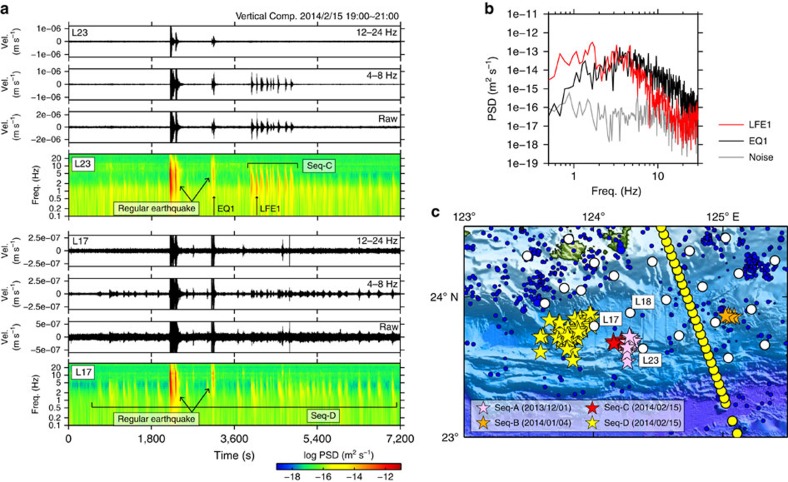
Seismic records and distribution of low-frequency earthquakes. (**a**) Bandpass-filtered and raw seismograms and spectrograms of velocity waveforms for sequence C at ocean bottom seismograph (OBS) L17 and for sequence D at OBS L23. Note that the LFEs are enriched in low-frequency (4–8 Hz) energy and depleted in high-frequency (12–24 Hz) energy although regular earthquakes show clear signals in the both frequency bands. (**b**) Comparison of power spectral density (PSD) of the selected low-frequency earthquake (LFE), regular earthquake and background noise. (**c**) Epicentres of LFEs (stars) and regular earthquakes (blue dots). Colours of the stars correspond to different sequences of LFEs (A–D). Yellow and white circles indicate the positions of OBSs for active and passive sources, respectively. Note that hypocenter locations of sequence C were accurately determined by manually picked arrival times, while only the epicentral locations were constrained by the envelope correlation method^27^ for sequence A, B and D.

Using the OBS network for passive source observation, we succeeded in detecting a total of 73 LFEs (Fig. 1). These LFEs have high amplitude in the predominant frequency of 10 Hz and below, and a longer duration than regular earthquakes (Fig. 2a,b). They consist of four swarms (sequence A, B, C and D in Fig. 2c). For sequence C including six LFEs, the hypocentral locations were determined using P- and S-wave arrival times manually picked from the waveform data (Supplementary Fig. 1). We applied the envelope correlation method^27^ to other events (sequence A, B and D in Fig. 2) since the initial onsets were too obscure to identify. In this method, the resolution in the depth direction is generally poor and we thus discuss only the epicentral distribution. When locating LFEs by using the envelop correlation method, we applied the bootstrap method to achieve more accurate estimates of hypocenters and their s.d. of LFEs (Supplementary Fig. 2). Location error of each LFE was obtained by taking the average of 50 s.d. of bootstrap samples. We only used events with the error of <0.1 degree in the latitudinal/longitudinal directions and most of them were within 0.005 degree in the both directions. All of the LFEs were located in the forearc region 40–80 km landward from the trench axis: on the map view, they overlap the locations of previously reported VLFEs^6^ and are distributed between the source regions of Yaeyama earthquake tsunami in 1771 close to the trench^11^ and repeating SSEs beneath the Ryukyu arc^5^ (Fig. 1).

**Figure 3:**
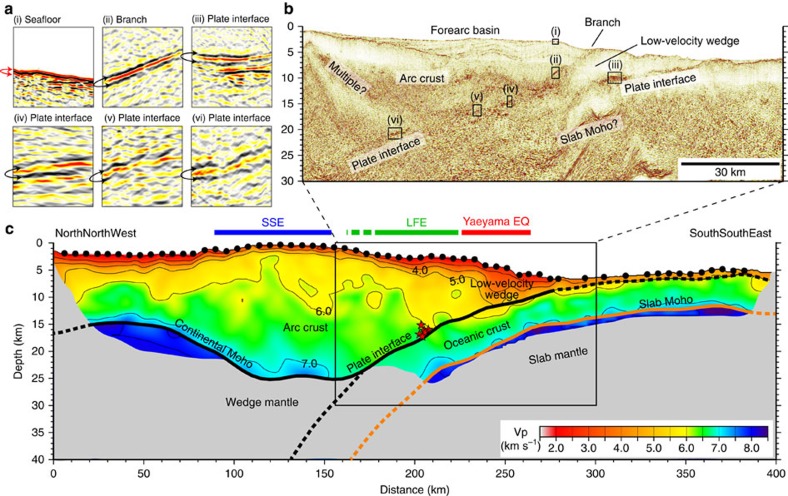
Seismic structure of the southern Ryukyu Trench. (**a**,**b**) Seismic reflection images. Note that a negative polarity at the branch (panel ii) and at several locations of the plate boundary (panels iii–vi) suggests a sudden velocity reduction probably due to high pore fluid pressures. (**c**) P-wave velocity model. Black dots show the locations of ocean bottom seismographs (OBSs). Areas with poor checkerboard recovery are masked. Thick black and orange lines indicate locations of the plate boundary/continental Moho and the Moho of the incoming oceanic plate, respectively. Dashed parts of these bold lines are not constrained by the data. Red stars in **c** indicate the locations of the LFEs (sequence C in Fig. 2). The source regions of the Yaeyama earthquake in 1771 (ref. 11) and slow slip events (SSEs)^5^ are shown above the P-wave velocity model by red and blue bars, respectively. The distribution of LFEs (green bar) bridges the spatial gap between these two regions.

**Figure 4:**
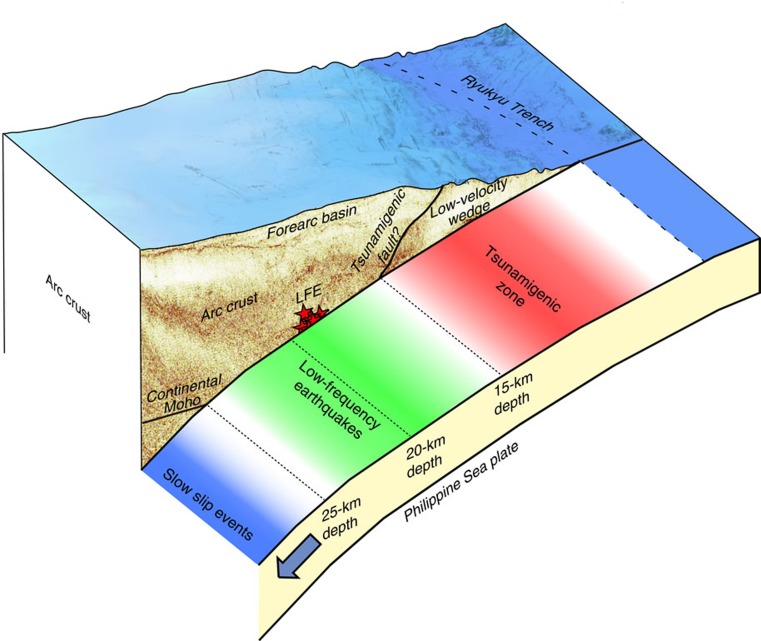
Perspective view of the subduction structure and distribution of low-frequency earthquakes (LFE) at the southern Ryukyu Trench. Red stars in the reflection image indicate the locations of the LFEs (sequence C in Fig. 2). In the shallow part of the subduction zone (red zone), a branch from the plate interface may have acted as a tsunamigenic fault. The LFEs we observed occurred at 15–18 km depths along the plate interface and their distribution (green zone) bridges the gap between the shallow tsunamigenic zone (red zone) and the deep slow slip region (blue zone).

The distribution of the LFEs is consistent with the plate geometry obtained by our active source seismic experiments. The MCS reflection data and refraction/wide-angle reflection data from the OBS records consistently reveal a clear image of the subducting Philippine Sea slab from the trench to ∼25 km depth (Fig. 3): The incoming plate starts to subduct at a low angle of ∼5° near the trench and increases its dip angle up to ∼15° with increase in depth. The events of sequence C were located at 15–18 km depths and close to the plate interface (Fig. 3). These LFEs are ∼60 km horizontally away from our seismic line, but the closer MCS section shows that the plate geometry does not vary significantly in the along-trench direction^28^ (Supplementary Fig. 3). We thus interpret that the LFEs of sequence C occurred along the plate interface. The LFEs in sequence D were located more landward compared with other activities (Figs 1 and 2). They are situated in the forearc basin where no major faults in the overriding plate which may host LFEs exist (Fig. 2 and Supplementary Fig. 3). Although the depths of sequence D were poorly constrained, they may also have occurred along the plate interface. Another possibility is that the LFEs occurred along strike-slip faults within the overriding plate^28^. We cannot fully exclude this possibility for sequence A due to its proximity to the strike-slip faults (Supplementary Fig. 2). Sequence B and most of the sequence D, however, are located far away from the faults. Judging from the location error of LFEs (also shown in Supplementary Fig. 2), this separation is significant. We thus suggest that at least sequence B, C and D are likely to occur along the plate boundary. The distribution of the LFEs seems to bridge the gap between the shallow tsunamigenic zone^11^ and the deep slow slip region^5^ (Fig. 1). This means that the plate interface in the southern Ryukyu subduction zone is dominated by slow earthquakes almost seamlessly and that a typical locked zone may be missing (Fig. 4). This independency of slow earthquakes with depth is unusual and significantly differs from the cases of strongly coupled subduction zone such as the Nankai Trough where LFEs and VLFEs are limited to a shallow plate interface and its surrounding faults at depths of less than 10 km and a deep transitional zone of over 30 km depth^19^.

### Tsunamigenic subduction faults

The Ryukyu subduction zone still has a high potential for large tsunami earthquakes and our seismic data set provides new observations that make a big step towards understanding the structural reasons for this. The most prominent structural feature we found is a ∼40-km-wide low-velocity zone at the seaward edge of the overriding plate (Fig. 3). This low-velocity wedge is bounded by the plate interface and another landward-dipping reflector branching from the plate interface at ∼15 km depth (MCS reflection section in Fig. 3). On the landward side of the branching reflector, subhorizontal reflectors separate the slope sedimentary sequences from the deeper unit (Fig. 3). The P-wave velocity model suggests that this boundary demarcates lower-velocity materials on the seaward side (Vp=2.0–5.0 km s^−1^) from a higher-velocity arc crust on the landward side (Vp=3.0–6.0 km s^−1^; Fig. 3). The branching reflector has a steeper angle of ∼15° and reaches the seafloor. Importantly, this branch overlaps a source region of the Yaeyama earthquake in 1771 and is consistent with the fault parameter estimated by the tsunami data (reverse faulting with dip angle of 12°)^11^. The reflection image shows that the sedimentary layer on the arc crust exhibits highly undulated structure (Fig. 3). This folding pattern is clearly visible from the shallowest layer to the top of the arc crust and thus suggests that the forearc region is still under a compressional stress regime. We therefore propose that a reverse fault motion along the branching fault is the most likely candidate for the source of the Yaeyama earthquake and tsunami. However, considering the uncertainty of the fault model of the Yaeyama earthquake^11^, we cannot fully exclude the possibility that the plate interface has played a role in generating this large earthquake and tsunami.

The structure of the incoming Philippine Sea plate is characterized as typical oceanic lithosphere: The crustal part is composed of a ∼1-km-thick upper layer with a rapid increase in velocity from 4.0 to 6.0 km s^−1^ (oceanic layer 2) and a thick lower layer of Vp=6.0–7.1 km s^−1^ with a more gradual velocity gradient (oceanic layer 3). However, the reflection image shows that the incoming plate has an extremely thin sedimentary layer on its top (Fig. 3). The poor sediment supply from the seaward side probably indicates that the sediments from the arc side significantly contribute to forming the low-velocity wedge. The horizontal scale of the low-velocity wedge (∼40 km) is significantly smaller than the accretionary prisms in the Nankai Trough^29^ and is equivalent to the forearc structure in the Japan Trench^30^. An important feature is that the landward tip of the low-velocity wedge extends ∼70 km from the trench axis and reaches a significant depth of at least 15 km (Fig. 3). This wedge shape is especially similar to one in the Japan Trench off Sanriku where the plate coupling is thought to be weak and a typical tsunami earthquake occurred in 1896 (ref. 30). A consideration is that sedimentary materials enriched in fluids are deeply dragged along the plate boundary and effectively extend a region of slow ruptures leading to tsunami earthquakes.

In the reflection image, an obvious negative polarity is observed at the branch and plate interface (panels ii–vi in Fig. 3), which ensures that there is a sudden velocity decrease across these interfaces. These low-velocity zones are too narrow to be resolved by travel time data of the OBS records. As for the polarity change at the branch, it may represent a velocity reduction from the overriding arc crust to the underthrust low-velocity wedge. Along the plate interface, the negative polarity is found not only beneath the low-velocity wedge but also at its deep portion where the LEFs occur. A plausible interpretation is that the reverse polarity corresponds to fault zones with high pore fluid pressures. We emphasize that at the southern Ryukyu Trench the plate interface exhibits a negative polarity at a wide range of depth from 5 to 22 km and that this feature is uncommon in strongly coupled subduction zone^31^. This structural evidence is consistent with the weak plate coupling in the Ryukyu subduction zone since trapped fluids with high pore pressures can weaken the fault zone by lowering effective normal stress^32^. Slow ruptures enhanced by fluids may be a controlling factor of LFEs, SSEs and potential tsunami earthquakes.

## Methods

### Data acquisition

The seismic experiments were carried out in November 2013, using the JAMSTEC research vessel *Kairei*. For recording passive events, we deployed 30 OBSs and 6 seismometers at onshore stations and recovered them after 3 months. For refraction studies, a tuned airgun array with a total volume of 7,800 cubic inches was towed at 10 m depth and fired every 200 m. The seismic data were recorded on 60 OBSs laid out along a 390-km-long profile with an average spacing of ∼6 km. Each unit contained a three-component seismometer and a hydrophone; the sampling interval was 4 ms. The MCS reflection data were obtained on the same line using a 6,000-m-long, 444-channel streamer cable. The shot interval was 50 m for the reflection survey.

### Locating LFEs

During the passive observation, we detected a total of 73 LFEs (Fig. 2). Some of the events (sequence C in Fig. 2) have a sharp onset of P-wave at the nearest OBS and S-wave onset at several stations (Supplementary Fig. 1). We located them using these arrival times and tomoFDD code^33^. The 3D velocity structure for location was derived from the final P-wave velocity model of the refraction analysis (Fig. 3) and the S-wave velocity was constructed by dividing the P-wave velocity by 1.73. We tested several damping parameters and confirmed that the choice did not change the result significantly. Otherwise (sequence A, B and D in Fig. 2) initial onsets were too obscure to identify. We applied the envelope correlation method^27^ for the vertical component of these events to measure differential travel times between stations based on a criterion that the correlation coefficient is larger than 0.85. Their hypocenters and s.d. were estimated by the bootstrap methods^34^ using 50 different data samples (Supplementary Fig. 2). For velocity structure, a constant S-wave velocity of 3.5 km s^−1^ was assumed.

### Data processing for OBS refraction data

All OBSs for refraction studies were positioned by travel times of direct water waves from airgun shots. For better picking, we applied 3–12 Hz bandpass filter and automatic gain control to the OBS records (Supplementary Fig. 4). The 2D P-wave velocity model was constructed using an iterative tomographic technique that incorporates travel times of refraction, wide-angle reflection and near-vertical reflection^35^. The number of travel times used in the calculation was 33,880 for first arrival, 3,953 for wide-angle reflection from the plate boundary and continental Moho, 2,411 for wide-angle reflection from the slab Moho and 74 for near-vertical MCS reflections, respectively. Uncertainties of travel time picks of first arrivals ranged from 30 to 60 ms depending on the offset and signal/noise ratio, while those of reflection arrivals were 60 ms for all offsets. Reliability of the obtained velocity model was evaluated by the data misfit and checkerboard resolution test (Supplementary Fig. 5).

### Data processing for MCS reflection data

We applied a standard data processing flow including trace header edit, trace edit, common midpoint binning at 6.25 m intervals, bandpass filter, datum correction, amplitude compensation, minimum phase conversion, predictive deconvolution, velocity analysis, normal moveout correction, mute and common midpoint stack and post-stack time migration. For time-to-depth conversion, we used the P-wave velocity model derived by the tomographic inversion (Fig. 3).

### Data availability

The data that support the findings of this study are available from the corresponding author on request.

## Additional information

**How to cite this article:** Arai, R. *et al*. Structure of the tsunamigenic plate boundary and low-frequency earthquakes in the southern Ryukyu Trench. *Nat. Commun.* 7:12255 doi: 10.1038/ncomms12255 (2016).

## Supplementary Material

Supplementary Information Supplementary Figures 1-5 and Supplementary References.
