# Supplementary material for: Structure of the tsunamigenic plate boundary and low-frequency earthquakes in the southern Ryukyu Trench
Source: Nat Commun. 2016 Jul 22;7:12255. doi: 10.1038/ncomms12255 (PMC4961848; doi:10.1038/ncomms12255)
Supplement: Supplementary Information — Supplementary Figures 1-5 and Supplementary References. [file ncomms12255-s1.pdf]

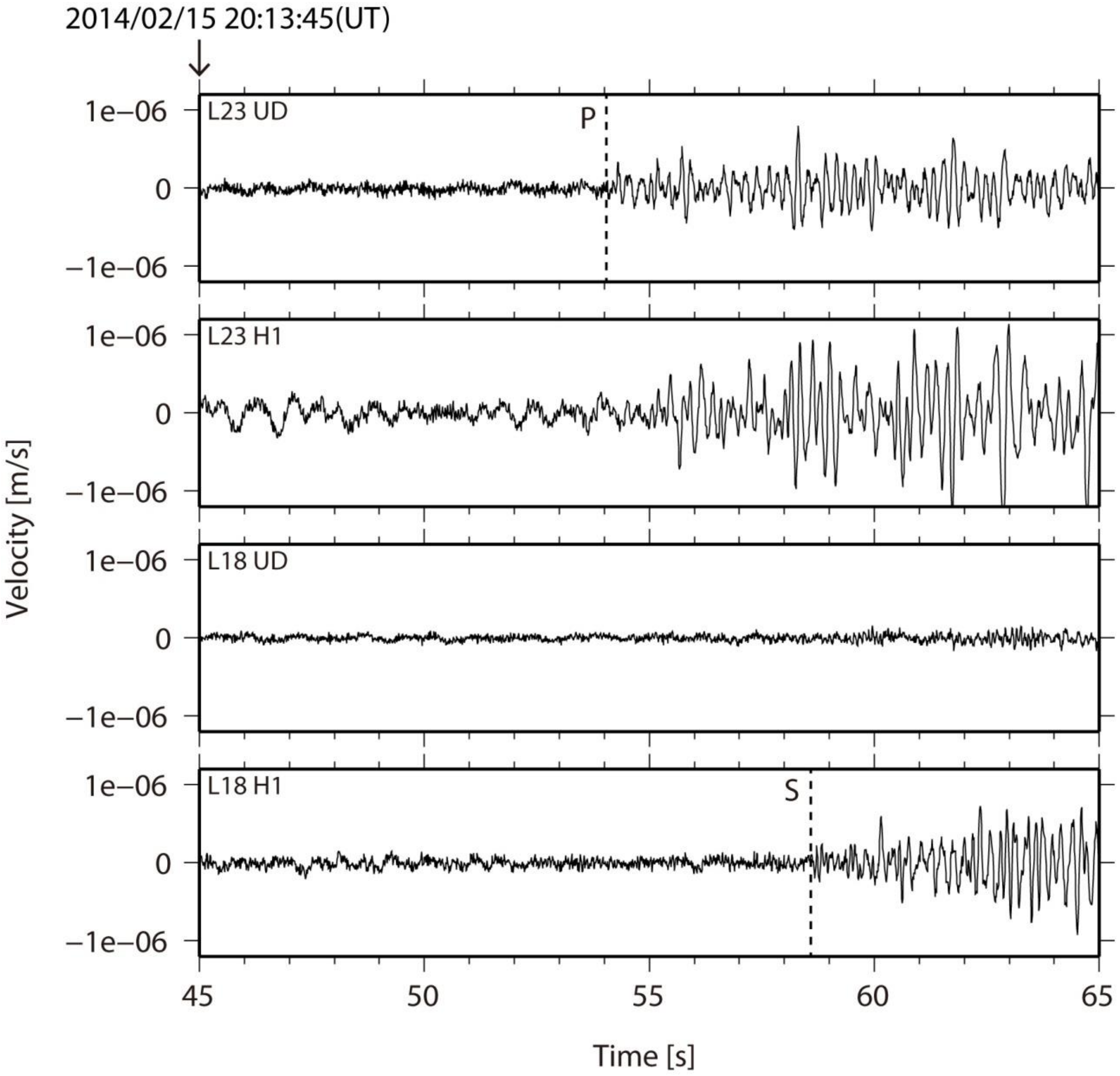

Supplementary Figure 1. Examples of raw seismograms of low-frequency earthquakes (LFEs) (sequence-C). Vertical and horizontal components of L23 and L18 from top to bottom. Dashed lines indicate P- and S-wave arrival times manually picked from the waveform data.

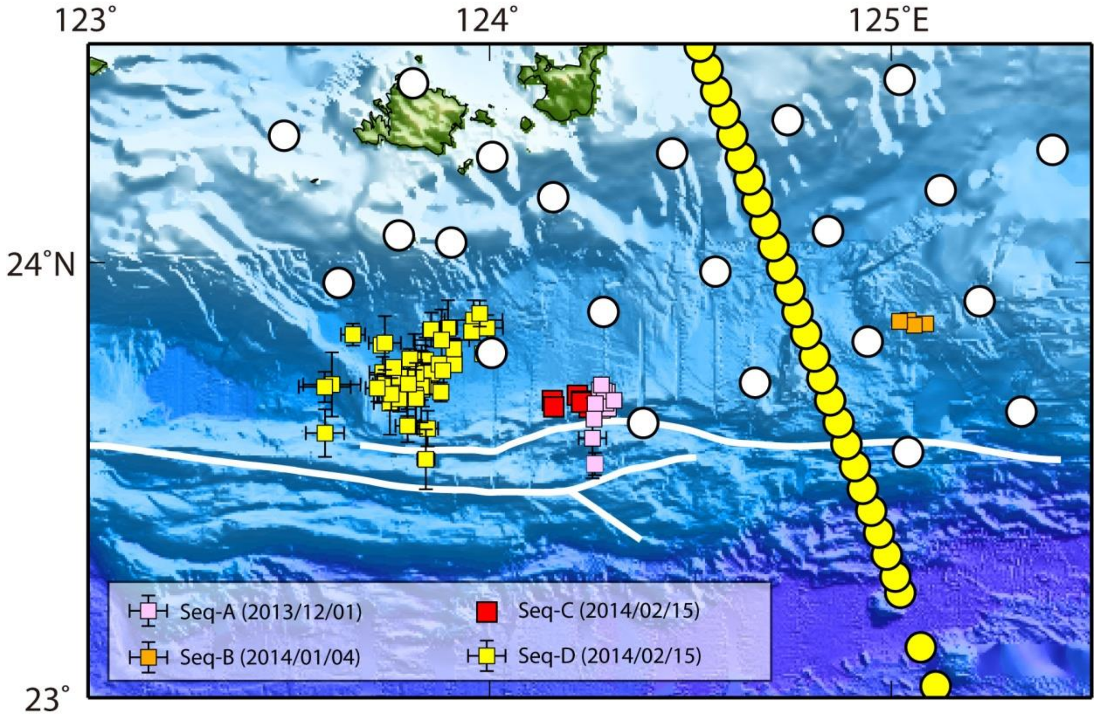

Supplementary Figure 2. Distribution of low-frequency earthquakes (LFEs) and their location errors. For estimating location errors of LFEs (sequence-A, -B, and –D), we calculated their standard deviations by using the bootstrap method[1] using 50 different traveltime data sets. Black bars attached to each event indicate 1 standard deviation in the longitudinal/latitudinal directions. Yellow and white circles indicate the positions of ocean-bottom seismometers (OBSs) for active and passive sources, respectively. White lines are potential strike-slip faults proposed by ref. 2. Note that sequence-B and most of the sequence-D do not overlap the potential strike-slip faults and this separation is ensured by the small location errors of LFEs. Sequence-D is located within the forearc basin where the seafloor is flat and no major fault is recognized in the reflection sections (Supplementary Figure 3).

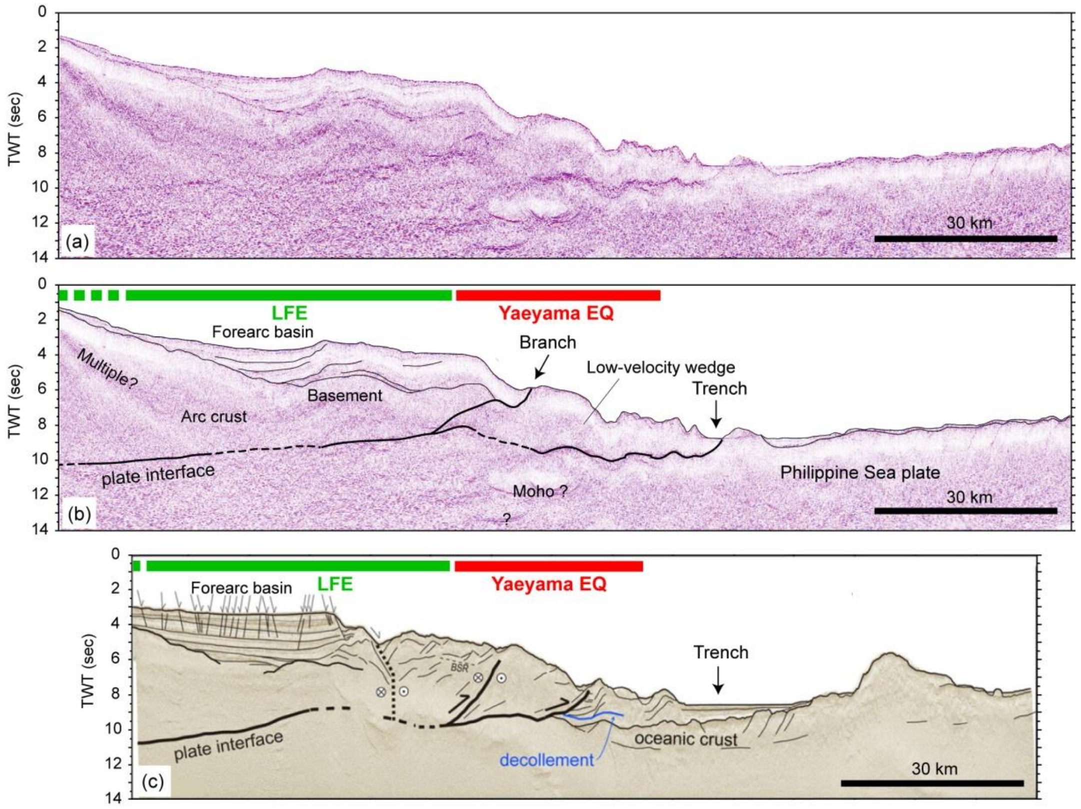

Supplementary Figure 3. Comparison of the forearc structure in the southern Ryukyu Trench. (a) Time migrated section of this study and (b) its interpretation. Panel (c) is from ref. 2. The location of the profile is shown in Figure 1. Color bars on the panels (b) and (c) represent the distribution of the Yaeyama earthquake[3] and low-frequency earthquakes (LFEs) constrained by this study.

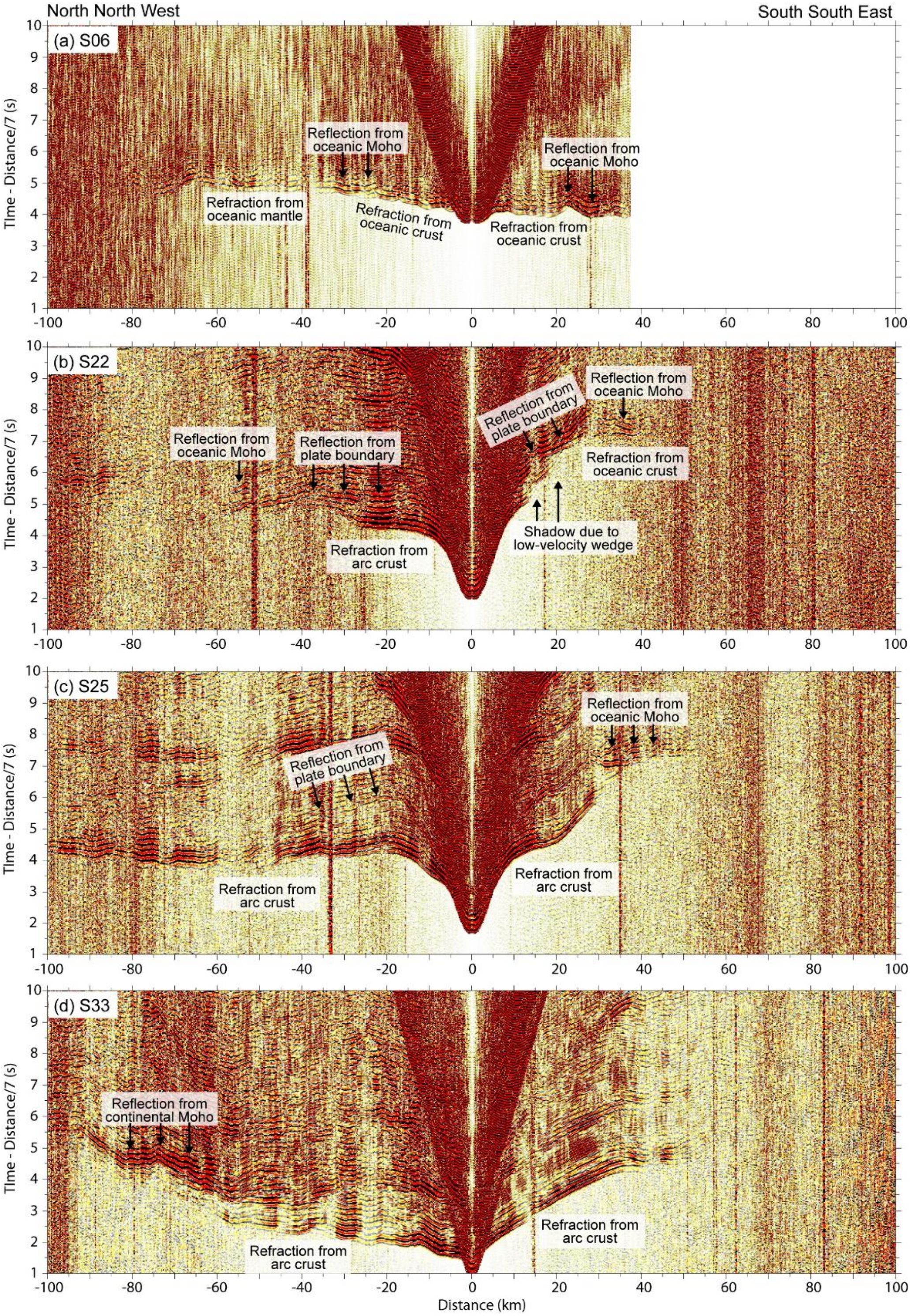

Supplementary Figure 4. Examples of the ocean bottom seismograph (OBS) records from the active source experiment. (a) S06. (b) S22. (c) S25. (d) S33. For each record, 3–12 Hz band-pass filter is applied. To compensate for geometrical

spreading, each trace is normalized by its offset. Locations of the OBSs are shown in Figure 1.

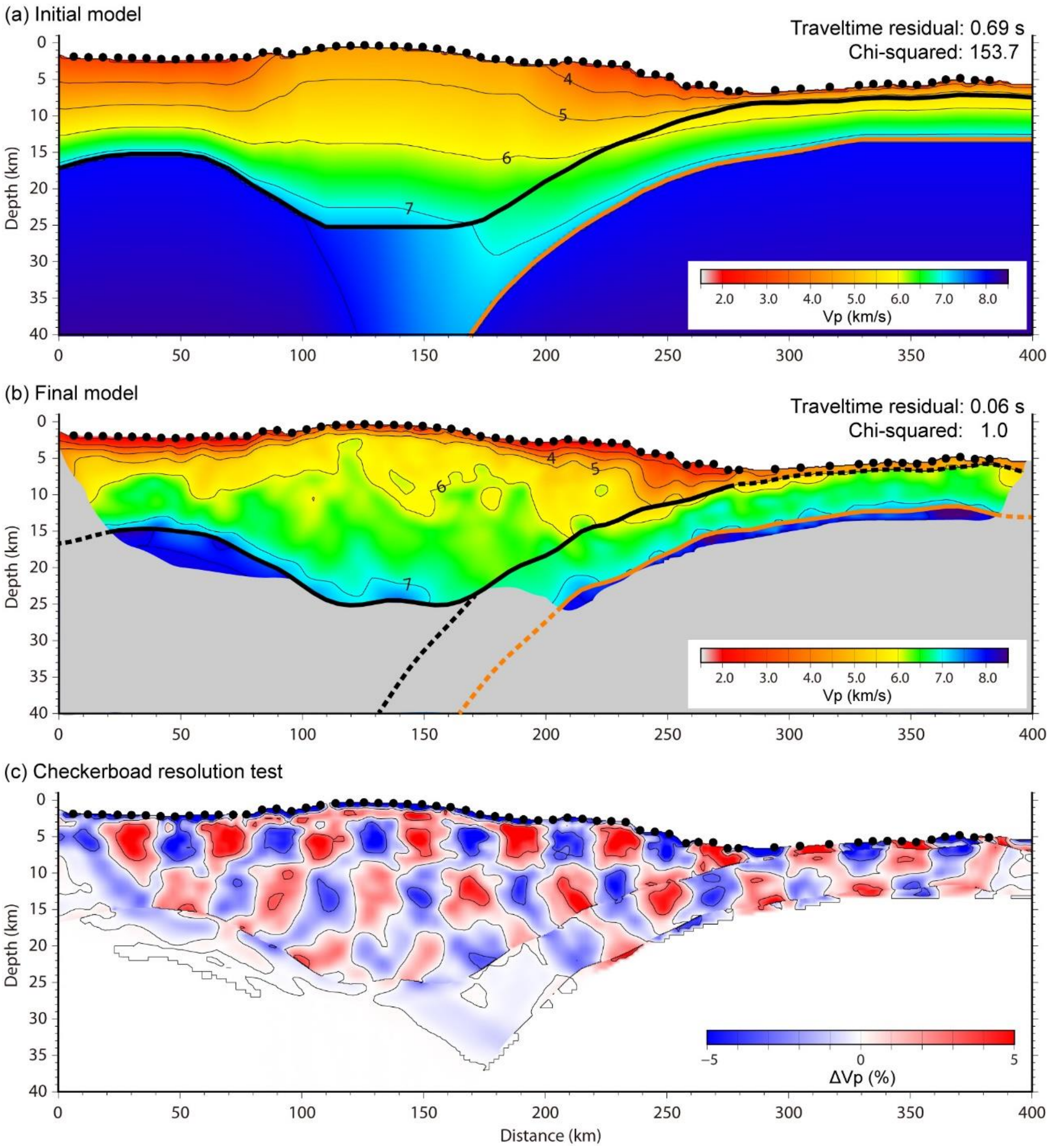

Supplementary Figure 5. P-wave velocity models and the result of checkerboard resolution test. (a) Initial model used in this study. To construct the initial model, we

referred to P-wave velocity models in the northern and middle Ryukyu subduction zone[4,5]. Thick black and orange lines indicate initial locations of the plate boundary/continental Moho and the Moho of the incoming oceanic plate, respectively. Black dots show the locations of ocean bottom seismographs (OBSs). (b) Final velocity model. The traveltime chi-squared residual converged 1.0 after 12 iterations. Areas with poor checkerboard recovery are masked. Dashed parts of the bold lines are not constrained by the data. (c) Result of checkerboard resolution test. For this test, the same ray paths were used as for the final solution. A reference model was created by adding sinusoidal anomalies with a maximum relative amplitude of 5 % to the final model. The vertical and horizontal sizes of the anomaly pattern are 7.5 km and 20 km, respectively.

Supplementary References

1. Efron, B. & Tibshirani, R. J. *An Introduction to the Bootstrap*. Boca Raton (CRC Press, 1994).
2. Hsu, S.-K., Yeh, Y.-C., Sibuet, J.-C., Doo, W.-B. & Tsai, C.-H. A mega-splay fault system and tsunami hazard in the southern Ryukyu subduction zone. *Earth Planet. Sci. Lett.* **362**, 99–107 (2013).

3. Nakamura, M. Fault model of the 1771 Yaeyama earthquake along the Ryukyu Trench estimated from the devastating tsunami. *Geophys. Res. Lett.* **36**, L19307 (2009).
4. Iwasaki, T. et al. Crustal and upper mantle structure in the Ryukyu Island Arc deduced from deep seismic sounding. *Geophys. J. Int.* **102**, 631-651 (1990).
5. Kodaira, S. et al. Crustal structure across the middle Ryukyu trench obtained from ocean bottom seismographic data. *Tectonophysics* **263**, 39-60 (1996).
